# Are the Two Human Papillomavirus Vaccines Really Similar? A Systematic Review of Available Evidence: Efficacy of the Two Vaccines against HPV

**DOI:** 10.1155/2015/435141

**Published:** 2015-08-25

**Authors:** Simona Di Mario, Vittorio Basevi, Pier Luigi Lopalco, Sara Balduzzi, Roberto D'Amico, Nicola Magrini

**Affiliations:** ^1^SaPeRiDoc Unit, Department of Primary Health Care, Regional Health Authority of Emilia-Romagna, Viale Aldo Moro 21, 40127 Bologna, Italy; ^2^Office of Chief Scientist, European Centre for Disease Prevention and Control (ECDC), 171 83 Stockholm, Sweden; ^3^Statistics Unit, Department of Diagnostic and Clinical Medicine and Public Health, University of Modena & Reggio Emilia, Via del Pozzo 71, 41100 Modena, Italy; ^4^Drug Evaluation Unit, WHO Collaborating Centre for Evidence Based Research Synthesis and Guidelines Development, Regional Health and Social Agency of Emilia-Romagna, Viale Aldo Moro 21, 40127 Bologna, Italy

## Abstract

*Background*. When the bivalent and the quadrivalent HPV vaccines were marketed they were presented as having comparable efficacy against cervical cancer. Differences between the vaccines are HPV types included and formulation of the adjuvant. *Method*. A systematic review was conducted to assess the efficacy of the two vaccines against cervical cancer. Outcomes considered were CIN2+, CIN3+, and AIS. *Results*. Nine reports (38,419 women) were included. At enrolment mean age of women was 20 years, 90% had negative cytology, and 80% were seronegative and/or DNA negative for HPV 16 or 18 (naïve women). In the TVC-naïve, VE against CIN2+ was 58% (95% CI: 35, 72); heterogeneity was detected, VE being 65% (95% CI: 54, 74) for the bivalent and 43% (95% CI: 23, 57) for the quadrivalent. VE against CIN3+ was 78% (95% CI: <0, 97); heterogeneity was substantial, VE being 93% (95% CI: 77, 98) for the bivalent and 43% (95% CI: 12, 63) for the quadrivalent. VE in the TVC was much lower. No sufficient data were available on AIS. *Conclusions*. In naïve girls bivalent vaccine shows higher efficacy, even if the number of events detected is low. In women already infected the benefit of the vaccination seems negligible.

## 1. Introduction

In the seventies, Dr. Harald Zur Hausen firstly postulated the link between human papillomaviruses (HPVs) and cervical cancer: studies to develop an anticancer vaccine followed. Approximately 70% of cervical cancers worldwide are associated with two high-risk HPV types (16/18) [1, 2] and almost 90% of genital warts are associated with two low-risk HPV types (6/11). Each year around 500,000 women develop invasive cervical cancer worldwide, with 83% of new cases and 85% of deaths occurring in developing countries [3, 4]. Risk factors associated with HPV infection are younger age at first coitus, higher number of sexual partners, smoking cigarettes, and history of herpes simplex virus infection [5, 6]. Some 75% of sexually active women develop a HPV infection [7], more frequently soon after their sexual debut: the majority of these infections (between 70% and 90%) spontaneously clear [8–10]. A minority progress from acute infection to cervical cancer, a process taking decades and going through precancerous lesions named cervical intraepithelial neoplasia (CIN) of increasing severity, from CIN1 to CIN3; spontaneous regression of the lesions is possible at any point [4, 8]. Incidence of genital warts is less precisely known, due to lack of data on the general population, but it is estimated to be around 1% [8].

Based on the results of five randomized controlled trials (RCTs) involving 40,000 women [11–17] two HPV vaccines entered the market. The quadrivalent vaccine against HPV6/11/16/18 was approved by the FDA and the EMA in 2006 [18, 19], whereas the bivalent vaccine against HPV16/18 was first approved in Europe in 2007 [20] and then in the USA in 2009 [21]. Soon after, several western countries such as the USA, Australia, and five European states [22] started national immunization campaigns targeting adolescent girls. The number of countries adopting the vaccines has since increased: in April 2014, 23 out of 29 European countries were reported to have implemented it [23], budgetary constraints being one relevant obstacle for the remaining countries. The primary target of HPV vaccination is adolescent girls aged 11 to 13 years, with some minor differences in national recommendations: in the USA routine vaccination is recommended at age 11 or 12 years with quadrivalent or bivalent vaccine for females and with quadrivalent vaccine for males in a 3-dose schedule during a 6-month interval [24]; in the UK HPV vaccine is recommended for girls under 15 years of age and consists in two injections spaced at least six and not more than 24 months apart. The vaccine is also recommended for men who have sex with men [25]. No prior assessment with Pap testing or screening for existing HPV infection is required.

Both vaccines contain human papillomavirus L1 self-assembling virus-like particles and are not infectious. Differences between the two vaccines are the number of HPV types included and formulation of the adjuvant (Table 1) possibly leading to different vaccine efficacy (VE) [36–38]. Head-to-head comparisons between the two vaccines are still exclusively based on immunogenicity [38, 39], although an immune correlate of protection has not yet been established [40].

Three meta-analyses, published when vaccines were marketed, showed comparably high efficacy of the two vaccines against precancerous lesions associated with HPV16/18 [30, 41, 42]. The meta-analyses subsequently published [43, 44] have confirmed the high efficacy of the vaccines against lesions associated with HPV16/18, but they did not provide information about VE against any cervical lesions irrespective of HPV type, nor did they discuss possible differences in terms of efficacy of the two vaccines. Thus, our systematic review that includes studies with longer follow-up aims to assess differences between the two vaccines from a public health perspective, considering all cervical lesions.

## 2. Methods

The systematic review was developed based on a prespecified protocol (protocol number FARM8N2ZFL) funded by the Italian Medicines Agency (AIFA) within a program of independent research on drugs [45]. The PRISMA (Preferred Reporting Items for Systematic Reviews and Meta-Analyses) statement guided the content and reporting of the review [46]. Published and unpublished RCTs comparing any of the two HPV vaccines versus placebo or any other control were considered for inclusion. We exclusively considered studies involving women, irrespective of age at enrolment.

As for protocol, primary outcome measures were cervical lesions (i.e., cervical cancer, CIN2, CIN3, and AIS) associated with any HPV type and cervical lesions exclusively associated with HPV16/18 occurring in three study populations: according to protocol population (ATP), the general population of vaccinated women (total vaccine cohort (TVC)) approximating all women regardless of status of HPV infection at vaccination, and a selected population of women seronegative for HPV16/18 and HPV DNA negative for 14 oncogenic HPV types, approximating the group of young adolescent girls targeted in the national immunization campaigns (TVC-naïve). In this paper only data related to cervical lesions associated with any HPV type occurring in the TVC and in the TVC-naïve population are reported, as the other data are not relevant from a public health perspective.

### 2.1. Literature Search

Trial identification: we searched the Cochrane Library (to Issue 3, 2014), MEDLINE (to March 2014), and EMBASE (to March 2014) using keywords and MeSH terms as reported in Annex 1 online, in combination with a highly sensitive filter for identifying RCT [47]. There were no language or time restrictions. Reference lists of relevant papers were also examined to identify additional studies. For unpublished RCTs, we searched the Internet for prepublication study presentations at conferences or meetings. Moreover experts in the field and vaccine manufacturers were contacted for further information (unpublished studies and single patient data). Clinical trial registers were searched for ongoing studies. Two review authors independently screened abstracts of potential studies and retrieved full articles for those deemed eligible.

### 2.2. Study Selection

Two reviewers carried out independent assessment of citations retrieved. When more than one publication reported the same trial, the one with a longer follow-up was selected. Reasons for exclusion were recorded. Quality of trials was assessed using the criteria outlined in the Cochrane Handbook [47] and included the assessment of (i) generation of the randomization sequence, (ii) quality of the allocation concealment, (iii) completeness of follow-up, and (iv) blinding of the outcome assessment. Based on quality assessment the risk of bias of the included trials was defined as low, high, or unclear. Differences in opinion were resolved through discussion involving a third author if needed.

### 2.3. Statistical Analysis

Two review authors extracted the data independently using a data extraction form. Statistical analyses were carried out using the STATA software version 11. For time to event data the hazard ratio (HR) was used as a measure of association. The results were summarized by using the inverse of variance method and the random effects model. Point estimates as well as their 95% confidence intervals (95% CI) were calculated and represented by the forest plot. Vaccine efficacy (VE) was calculated as VE(%) = (RU − RV)/RU × 100, where RU is the rate of disease in the unvaccinated and RV is the rate in the vaccinated [48]. The equation can be rewritten to use the HR in the following way: VE(%) = (1 − HRv/u) × 100, where HRv/u is the HR of the vaccinated versus the unvaccinated. Heterogeneity among studies was assessed using the *I* ^2^ statistics [47].

### 2.4. Subgroup Analyses

The following prespecified subgroup analyses were planned:

- Geographical areas (Europe, Africa, Asia, North America, and South and Central America): the literature suggests that prevalence and circulation of HPV high risk types varies according to geographical areas [49–51].
- Vaccine type (bivalent, quadrivalent): data on VE can be influenced by type of vaccine used as differences between the two formulations could be not negligible.

## 3. Results

### 3.1. Selection of Studies

**Figure 1:**
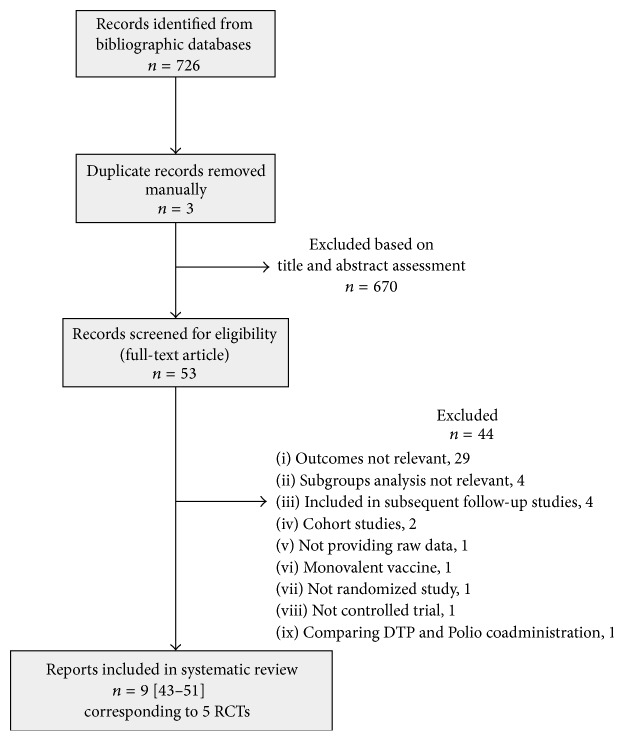
Study selection flowchart.

Study identification and selection process is outlined in Figure 1. Of the 726 records initially identified, 3 were duplicates and 670 were excluded based on title and abstract assessment. The most common reasons for exclusion were the following: reports were not RCTs, did not assess the outcome of interest, were not related to oncogenic HPV or to vaccine administration, or were studies reporting exclusively laboratory or immunogenicity data. We assessed the full text of 53 articles and excluded 44 (reasons reported in Figure 1). Thus nine reports [26–29, 31–35], corresponding to five registered protocols [52–56], were included in the systematic review: in three trials (20,797 women) the bivalent vaccine was used (PATRICIA trial being the larger study) [27–29] and in two trials (17,622 women) the quadrivalent vaccine was assessed (FUTURE I and FUTURE II trials) [26]. Five publications reporting subset of data of the above-mentioned trials relating to specific geographical areas were also included [31–35].

The data of the phase III trials included in our meta-analysis have a mean follow-up of 4 years for the bivalent trial [27] and 3.6 years for the quadrivalent trial [26], longer than that of the systematic reviews with meta-analysis published up to now.

### 3.2. Quality of Included Trials and Characteristics of Trial Participants

Characteristics of the trials are summarized in Table 2.

Studies were all double blind RCTs, with an adequate sample size. They were all manufacturer-sponsored. Eligible participants were healthy, not pregnant women aged between 15 and 26 years, with 6 or less lifetime sexual partners and no history of abnormal Pap smear at enrolment. Almost 90% of women had normal cytology at study entry. Risk of bias was low in 4 out of 5 trials: generation of the randomization sequence, quality of allocation concealment, completeness of follow-up, and blinding of outcome assessment were adequate. Risk of bias was unclear (generation of the randomization sequence and allocation concealment were not described) in one smaller trial conducted in Japan [29]; as the Japanese trial provided only data on CIN2+ lesions exclusively associated with HPV16/18, its results are not reported here. Characteristics of the women enrolled in the trials used in this meta-analysis are reported in Table 3.

### 3.3. Pooling of the Data

Pooling of the data was possible for CIN2+ [26–28], CIN3+ [26, 27], and AIS [26, 28]. Data for each outcome are presented below for the TVC and the TVC-naïve cohorts.

**Figure 2:**
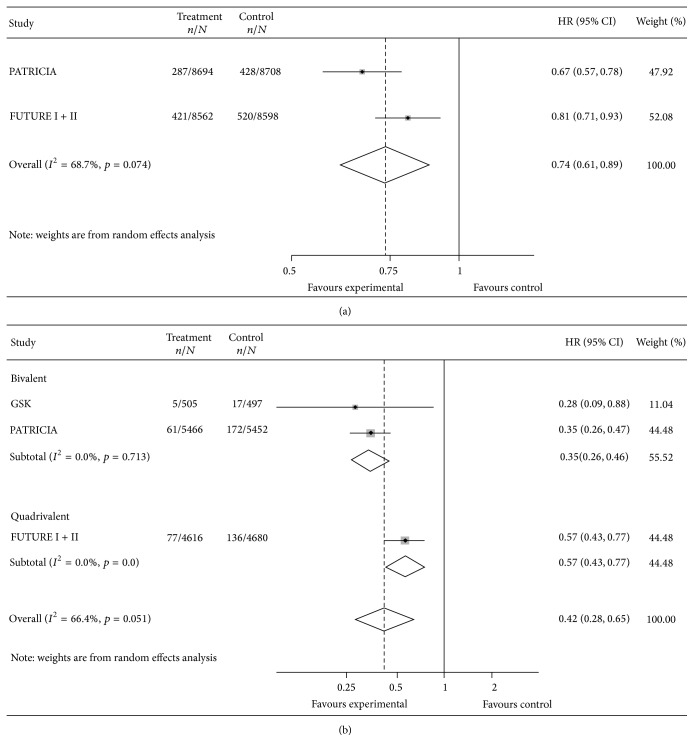
Vaccine efficacy against CIN2+ lesions, in total vaccine (a) and total vaccine naïve cohort (b), any HPV type.

*CIN2+*. The pooled HRs for CIN2+ lesions associated with any type of HPV in the TVC and in the TVC-naïve are reported in Figure 2. Corresponding values of VE were 26% (95% CI: 11, 39) in the TVC [26, 27] and 58% (95% CI: 35, 72) in the TVC-naïve [26–28]. Results suggested substantial heterogeneity among bivalent and quadrivalent vaccines: *I* ^2^ test was 68.7% and 66.4% in the TVC and in the TVC-naïve, respectively.

**Figure 3:**
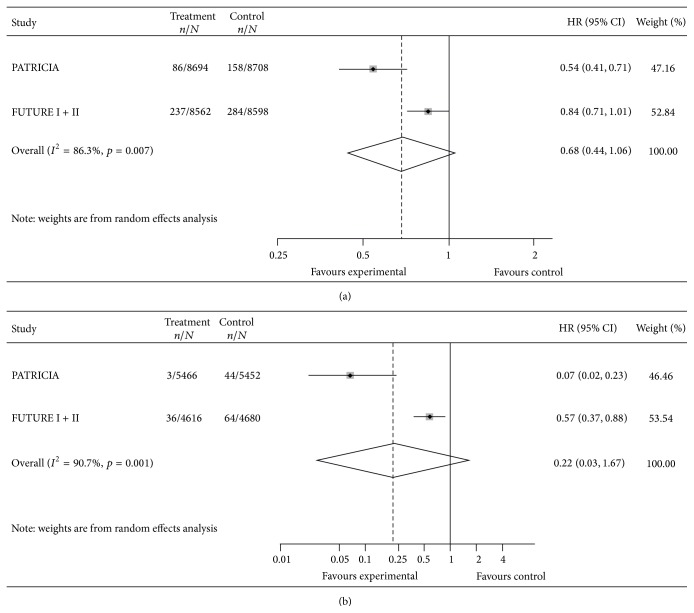
Vaccine efficacy against CIN3+ lesions, in total vaccine (a) and total vaccine naïve cohort (b), any HPV type.

*CIN3+.* The pooled HRs for CIN3+ lesions associated with any type of HPV in the TVC and in the TVC-naïve are reported in Figure 3. Corresponding values of VE were 32% (95% CI: <0, 56) in the TVC [26, 27] and 78% (95% CI: <0, 97) in the TVC-naïve [26, 27]. Results suggested substantial heterogeneity among bivalent and quadrivalent vaccines: *I* ^2^ test was 86.3% and 90.7% in the TVC and in the TVC-naïve, respectively.

*AIS*. The pooled HR for lesions associated with any type of HPV was 0.31 (95% CI: 0.14–0.70) in the TVC [26, 27], corresponding values of VE being 69% (95% CI: 30, 86).

AIS cases in the TVC-naïve cohort were zero in the vaccine group and ten in the placebo; thus only an approximate estimate of the efficacy was possible. The pooled HR for lesions associated with any type of HPV was 0.01 (95% CI: 0.01–0.22) resulting in a VE of 99% (95% CI: 78, 99) [26, 27]. For all the comparisons *I* ^2^ suggested low heterogeneity.

### 3.4. Analysis by Geographical Area

Although formally required by the scientific advisory unit of ECDC in Stockholm, the two manufacturers did not provide single patient data (the authors received only partial data unsuitable for the analysis from GSK and no answer at all from Sanofi Pasteur MSD).

Five published papers [31–35] reported data according to geographical areas. Pooling of the data was not appropriate since geographical areas definition differed (Table 4).

## 4. Discussion

Since their introduction into the market, the effectiveness of the two vaccines against cervical cancer, based on first published data [11–17], has been subject of debate. Enthusiastic positions assumed that if vaccines are immunogenic and prevent infections associated with HPV16/18 they also prevent cervical cancer and therefore should be widely used [57–59]. Uncertainty was related to the following issues: correlation between immune response and clinical outcomes, need for a booster dose, replacement with other oncogenic strains, and possible reduction of Pap-test screening among the vaccinated [60–65].

Our systematic review highlights that, for precancerous lesions (CIN), the only available proxy of cervical cancer and heterogeneity among pooled studies is substantial (Figures 2 and 3): the bivalent vaccine shows higher efficacy against precancerous lesions. We focus our comments on the TVC-naïve cohort, as VE in the TVC is confirmed to be much lower and HPV vaccination is not universally offered to women already sexually active and data on AIS are too sparse to make sensible comments. For CIN2+ lesions estimates of efficacy of the two vaccines in the TVC-naïve cohort differ but the wide limits of the confidence intervals partially overlap (VE 65%; 95% CI: 54, 74 for the bivalent and VE 43%; 95% CI: 23, 57 for the quadrivalent), whereas for CIN3+ lesions estimates of efficacy largely differ and the limits of the confidence intervals do not overlap (VE 93%; 95% CI: 77, 98 for the bivalent and VE 43%; 95% CI: 12, 63 for the quadrivalent). However, due to limited number of patients with lesions detected, leading to wide confidence intervals of our estimates of effect, our conclusions should be interpreted with caution. The heterogeneity observed might be due to higher efficacy of the bivalent vaccine against cervical cancer possibly related to the specific adjuvant used (ASO4 adjuvant system), as suggested in two recent immune response head-to-head studies that consistently showed a higher neutralizing antibody production [38, 39] and a higher CD4+ T cell response [38] in bivalent than in quadrivalent vaccine recipients. Heterogeneity can also be due to baseline differences between the populations enrolled in the two trials, although such differences were not reported in the two trials (Table 3). However, misclassification of naïve women cannot be ruled out since the two manufacturers used different laboratory tests to measure immune response and to identify naïve girls. In fact cLIA test was used in the FUTURE trials whereas ELISA test that has a higher sensitivity than cLIA [66] was used in the PATRICIA trial. Moreover, data are often differently and poorly reported in the published trials [26–29, 31–35]; thus our ability to make meaningful comparisons and further analysis (e.g., assess the possible effect modification by smoking status or age) is hindered. We asked the manufacturers to provide individual patient data, but we did not receive a positive answer.

Another relevant point is that the length of the follow-up in the trials assessed seems insufficient to detect information relevant to the public and to policy-makers: as time interval from HPV infection to cervical cancer development is approximately 20 years, all information gathered in a much shorter period of time is not conclusive. Nevertheless the PATRICIA trial has a planned follow-up of 4 years [27] and longer follow-up data on bivalent vaccine are only available for 436 Brazilian women enrolled in a previous phase II trial [67]. We will have more information in 2020, when the results of the Finnish study that extended the follow-up for Finnish girls enrolled in the PATRICIA study will be published [68] and when the extension studies for the FUTURE II trial assessing the quadrivalent vaccine will also be available. In the meantime an open debate in this respect is urgently needed: national health agencies should set up a surveillance system to provide data on actual vaccines efficacy in the field and to allow international comparisons. At the moment, this comparison is not possible and we still do not know how to choose between the two vaccines [69]. Contrary to what is previously reported in other meta-analyses [30, 41–44], our systematic review suggests that the quadrivalent and the bivalent vaccines differ in terms of efficacy. This could be attributable to the different adjuvants contained in the two vaccines. Such difference in efficacy has not been widely recognized by national health agencies. For example, in Italy HPV vaccines are chosen and purchased through tendering schemes organized by regional health authorities that are based on the lowest price [70].

Apart from cervical cancer prevention, quadrivalent vaccine is known to effectively prevent genital warts [71, 72], whereas the bivalent vaccine can only marginally impact on these benign but distressing lesions [73]. The UK and a few Italian regions have recently substituted the bivalent with the quadrivalent vaccine, assuming comparable efficacy of the two vaccines against cervical cancer and giving an additional value to the activity against genital warts of the quadrivalent vaccine [10, 74]. Unfortunately, it is not possible to anticipate the consequences of the UK's and Italian choice: if different vaccine efficacy of the bivalent and the quadrivalent vaccine is going to be confirmed, lack of equivalence in terms of cervical cancer prevention could become an issue. The availability in the coming years of new broader spectrum HPV prophylactic vaccines could provide more insight into the current debate [75, 76].

## 5. Conclusions

In conclusion, we acknowledge that this systematic review has some limitations due to a low number of women with events and a high heterogeneity among trials that suggest caution in the interpretation of results. However, our conclusions are consistent with those from recent immunogenicity head-to-head studies [38, 39] and that provides strength to our interpretation. Our systematic review suggests that after nine years since HPV vaccines were introduced, their estimates of efficacy seem to diverge over time. The decision to consider the two vaccines similar in terms of cervical cancer prevention seems challenged by our longer term follow-up analyses. This might have implications for policy and pragmatic choices and deserves an open and comprehensive discussion. Moreover, international comparisons of the actual effectiveness of the two vaccines used in the field can add valuable information.

Finally, regulatory agencies should encourage the pharmaceutical companies to provide data across trials to assess all relevant outcomes in a comparable way, to reduce uncertainty, and to support health policy-makers that have to choose between alternative options [77, 78].

## Supplementary Material

The supplementary material shows the search strategy that was adapted for each database searched.

## Figures and Tables

**Table 1:** Characteristics of bivalent and quadrivalent HPV vaccine.

	Quadrivalent vaccine	Bivalent vaccine
Commercial name	Gardasil/Silgard	Cervarix

Manufacturer	Sanofi Pasteur MSD SNC	GlaxoSmithKline Biologicals S.A.

HPV types	HPV 6 L1 protein 20 *μ*g	HPV 16 L1 protein 20 *μ*g
	HPV 11 L1 protein 40 *μ*g	HPV18 L1 protein 20 *μ*g
	HPV 16 L1 protein 40 *μ*g	
	HPV 18 L1 protein 20 *μ*g	

Common characteristics	L1 protein in the form of noninfectious virus-like particles produced by recombinant DNA technology	

Differences in cellular culture	Yeast cells (*Saccharomyces cerevisiae *CANADE 3C-5 (strain 1895))	Hi-5 Rix4446 cells derived from *Trichoplusia ni* using a Baculovirus expression system

Differences in adjuvant	Amorphous aluminium hydroxyphosphate sulfate adjuvant, 225 *μ*g	AS04 adjuvant system composed of aluminium hydroxide and 3-O-desacyl-4′-monophosphoryl lipid A, 50 *μ*g

Therapeutic indications	Gardasil is a vaccine for use from the age of 9 years for the prevention of (i) premalignant genital lesions (cervical, vulvar, and vaginal) and cervical cancer causally related to certain oncogenic human papillomavirus (HPV) types; (ii) genital warts (condyloma acuminata) causally related to specific HPV types.	Cervarix is a vaccine for use from the age of 9 years for the prevention of (i) premalignant cervical lesions and cervical cancer causally related to certain oncogenic human papillomavirus (HPV) types.

Efficacy data leading to registration	95.2% (87.2, 98.7)^1^	90.4% (53.4, 99.3)

Efficacy data in the latest publication	43% (13, 63)^3^	93% (79, 99)^4^

Mean follow-up of phase III trials	3.6 years^1^	4 years^2^

^1^Vaccine efficacy against CIN/AIS associated with vaccine related HPV in the TVC-naïve from EMA registration data [19].

^2^Vaccine efficacy against CIN2+ associated with vaccine related HPV in the TVC-naïve from EMA registration data [20].

^3^Vaccine efficacy against any CIN3 in the TVC-naïve from Muñoz et al. 2010 [26].

^4^Vaccine efficacy against any CIN3+ in the TVC-naïve from Lehtinen et al. 2012 [27].

**Table 2:** Characteristics of the five trials selected.

Study [reference]	Protocol	Number of study sites	Countries included	Year of study enrolment	Funding source	Inclusion criteria		Exclusion criteria	Vaccine type^*∗*^	Control	Women enrolled (TVC)	Women assessed (TVC)	Length of follow-up
						Age years	Sexual partners	Any of the following					
GSK [11, 12, 28]	NCT00120848 Phase II trial	27	Brazil, Canada, and USA	2001	GSK	15–25	≤6	Abnormal cervical cytology, HPV16/18 seropositivity, DNA positivity for 14 oncogenic HPV types, history of abnormal Pap test.	B	Aluminium containing placebo	560 V 553 P	505 V 497 P	5.9 years (average) 6.4 years (maximum)

PATRICIA [13, 27]	NCT00122681 Phase III trial	135	14 (Asia-Pacific, Europe, Latin America, and North America)	2004-2005	GSK	15–25	≤6	History of abnormal Pap test, pregnancy or breastfeeding, chronic disease, autoimmune disease, immunodeficiency.	B	HAV vaccine	9319 V 9325 P	8694 V 8708 P	4 years (average)

Konno et al. [29]	NCT00316693 Phase II trial	13	Japan	2006	GSK	20–25	Not specified	History of abnormal Pap test or genital warts, pregnancy, previous vaccination with HPV or HAV vaccine, MPL administration, hepatitis A infection.	B	HAV vaccine	519 V 521 P	501 V 501 P	13.6 months in Konno 2010

FUTURE I [16, 26, 30]	NCT00092521 Phase III trial	62	16 (Asia-Pacific, Europe, and America)	2001–2003	Merck	16–24	≤4	History of abnormal Pap test or genital warts, pregnancy, being not healthy.	Q	Aluminium containing placebo	2723 V 2732 P	7980 V 7236 P Follow-up FUTURE I + FUTURE II trial	3.6 years (average) 4.9 years (maximum)

FUTURE II [17, 26, 30]	NCT00092534 Phase III trial	90	13 (as above)	2002-2003	Merck	15–26	≤4	History of abnormal Pap test or genital warts, pregnancy, being not healthy.	Q	Aluminium containing placebo	6087 V 6080 P	As above	As above

^*∗*^B: bivalent vaccine; Q: quadrivalent vaccine.

**Table 3:** Baseline characteristics of women enrolled in the four studies presented in the meta-analysis.

Study [reference]	Age mean (SD)		HPV 16 positivity at enrolment (%)				HPV 18 positivity at enrolment (%)				Lifetime number of sexual partners (median)			Smoking status (%)		*Chlamydia trachomatis * (%)		Hormonal contraceptive use (%)		Cytological abnormality at entry (%)	
			DNA		Serology		DNA		Serology												
	V	C	V	C	V	C	V	C	V	C		V	C	V	C	V	C	V	C	V	C
GSK [11, 12, 28]	20 ± 3	21 ± 3	NR	NR	NR	NR	NR	NR	NR	NR		NR	NR	NR	NR	NR	NR	NR	NR	NR	NR
PATRICIA [13, 27]	20 ± 3	20 ± 3	14	14	2	1	11	10	1	1	*0* ^*∗*^	4%	4%	30	30	6	5	59	61	10	9
											*1* ^*∗*^	74%	74%								
											*2* ^*∗*^	14%	15%								
											*≥3* ^*∗*^	8%	8%								
FUTURE I [16, 26, 30]	20 ± 2	20 ± 2	9	8	12	12	3	3	3	3		2	2	26	26	4	5	58	57	11	12
FUTURE II [17, 26, 30]	20 ± 2	20 ± 2	9	9	11	11	4	4	4	4		2	2	NR	NR	4	4	59	60	12	11

V: vaccine group; C: control group; NR: not reported.

^*∗*^Results are presented as percentage of women stratified by number of sexual partners.

**Table 4:** Vaccine efficacy against CIN2+ in different geographical regions (Latin America, Asia-Pacific, and Europe).

Study [reference]	Vaccine type^*∗*^	Geographical region	Protocols included	Women enrolled	Outcomes assessed	Lesion *n*/*N*		VE% (95% CI)
						Vaccine	Control	
Perez et al. [31]	Q	Latin America	V501-007; V501-0013; V501-0015; V501-0016; V501-0018	6400	CIN2+ HPV 6/11/16/18 in			
					*ATP cohort *	1/2415	21/2377	95.3 (71.0, 99.9)
					*TVC-naïve *	3/2671	26/2681	88.5 (62.5, 97.8)
					*TVC *	45/2718	67/2725	33.1 (1.0, 55.2)

Tay et al. [32]	Q	Asia-Pacific	V501-0013; V501-0015; V501-0016	814	CIN2+ HPV 6/11/16/18 in			
					*ATP cohort *	0/302	5/312	100.0 (−12.4, 100.0)

Majewski et al. [33]	Q	Europe	V501-007; V501-0013; V501-0015; V501-0016	9265	CIN2+ HPV 6/11/16/18 in			
					*ATP cohort *	0/4043	38/4043	100.0 (89.9, 100.0)
					CIN2+ any type in			
					*TVC-naïve *	23/2470	54/2527	56.6 (28.0, 74.6)

Barr et al. [34]	Q	North America	V501-005; V501-007; V501-0013; V501-0015; V501-0016	5996	CIN2+ HPV 16/18 in			
					*TVC-naïve *	0/2100	35/2116	100.0 (89.0, 100.0)
					*TVC *	19/2313	57/2356	66.4 (42.7, 81.1)
					CIN2+ any type in			
					*TVC *	72/2313	108/2356	33.0 (8.9, 51.0)

de Carvalho et al. [35]	B	Brazil	NCT00689741 HPV001; NCT00120848 HPV007; NCT00518336 HPV023	431	CIN2+ HPV 16/18 in			
					*TVC *	0/219	3/212	100.0 (−129.8, 100.0)
					CIN2+ any type in			
					*TVC *	5/219	8/212	40.6 (−106.0, 84.7)

CIN: cervical intraepithelial neoplasia; ATP: according to protocol; TVC: total vaccine cohort.

^*∗*^Vaccine type Q = quadrivalent; B = bivalent.

## References

[1] Clifford G., Franceschi S., Diaz M., Muñoz N., Villa L. L. (2006). Chapter 3: HPV type-distribution in women with and without cervical neoplastic diseases. *Vaccine*.

[2] Sargent A., Bailey A., Almonte M. (2008). Prevalence of type-specific HPV infection by age and grade of cervical cytology: data from the ARTISTIC trial. *British Journal of Cancer*.

[3] Sankaranarayanan R., Ferlay J. (2006). Worldwide burden of gynaecological cancer: the size of the problem. *Best Practice and Research: Clinical Obstetrics and Gynaecology*.

[4] Schiffman M., Castle P. E. (2005). The promise of global cervical-cancer prevention. *The New England Journal of Medicine*.

[5] Moscicki A.-B., Hills N., Shiboski S. (2001). Risks for incident human papillomavirus infection and low-grade squamous intraepithelial lesion development in young females. *The Journal of the American Medical Association*.

[6] Vinodhini K., Shanmughapriya S., Das B. C., Natarajaseenivasan K. (2012). Prevalence and risk factors of HPV infection among women from various provinces of the world. *Archives of Gynecology and Obstetrics*.

[7] Koutsky L. (1997). Epidemiology of genital human papillomavirus infection. *The American Journal of Medicine*.

[8] Frazer I. H., Cox J. T., Mayeaux E. J. (2006). Advances in prevention of cervical cancer and other human papillomavirus-related diseases. *Pediatric Infectious Disease Journal*.

[9] Ronco G., Giorgi-Rossi P., Carozzi F. (2010). New Technologies for Cervical Cancer screening (NTCC) Working Group. Efficacy of human papillomavirus testing for the detection of invasive cervical cancers and cervical intraepithelial neoplasia: a randomised controlled trial. *The Lancet Oncology*.

[10] Oakeshott P., Aghaizu A., Reid F. (2012). Frequency and risk factors for prevalent, incident, and persistent genital carcinogenic human papillomavirus infection in sexually active women: community based cohort study. *British Medical Journal*.

[11] Harper D. M., Franco E. L., Wheeler C. (2004). Efficacy of a bivalent L1 virus-like particle vaccine in prevention of infection with human papillomavirus types 16 and 18 in young women: a randomised controlled trial. *The Lancet*.

[12] Harper D. M., Franco E. L., Wheeler C. M. (2006). Sustained efficacy up to 4·5 years of a bivalent L1 virus-like particle vaccine against human papillomavirus types 16 and 18: follow-up from a randomised control trial. *The Lancet*.

[13] Paavonen J., Jenkins D., Bosch F. X. (2007). Efficacy of a prophylactic adjuvanted bivalent L1 virus-like-particle vaccine against infection with human papillomavirus types 16 and 18 in young women: an interim analysis of a phase III double-blind, randomised controlled trial. *The Lancet*.

[14] Villa L. L., Costa R. L. R., Petta C. A. (2005). Prophylactic quadrivalent human papillomavirus (types 6, 11, 16, and 18) L1 virus-like particle vaccine in young women: a randomised double-blind placebo-controlled multicentre phase II efficacy trial. *Lancet Oncology*.

[15] Villa L. L., Costa R. L. R., Petta C. A. (2006). High sustained efficacy of a prophylactic quadrivalent human papillomavirus types 6/11/16/18 L1 virus-like particle vaccine through 5 years of follow-up. *British Journal of Cancer*.

[16] Garland S. M., Hernandez-Avila M., Wheeler C. M. (2007). Quadrivalent vaccine against human papillomavirus to prevent anogenital diseases. *The New England Journal of Medicine*.

[17] FUTURE II Study Group (2007). Quadrivalent vaccine against human papillomavirus to prevent high-grade cervical lesions. *The New England Journal of Medicine*.

[18] FDA http://www.fda.gov/BiologicsBloodVaccines/Vaccines/ApprovedProducts/ucm111283.htm.

[19] AAVV (2006). *Scientific Discussion. Document for Registration of Gardasil to EMEA*.

[20] AAVV http://www.ema.europa.eu/docs/en_GB/document_library/EPAR_-_Scientific_Discussion/human/000721/WC500024636.pdf.

[21] FDA (2009). *Clinical Review of Biologics License Application for Human Papillomavirus 16, 18 L1 Virus Like Particle Vaccine, AS04 Adjuvant-Adsorbed (Cervarix)*.

[22] King L. A., Lévy-Bruhl D., O'Flanagan D. (2008). VENICE Country Specific Gate Keepers and Contact Points. Introduction of human papillomavirus (HPV) vaccination into national immunisation schedules in Europe: results of the VENICE 2007 survey. *Euro Surveillance*.

[23] European Centre for Disease Prevention and Control (2014). *HPV Vaccination in EU Countries: Review of New Evidence*.

[24] Markowitz L. E., Dunne E. F., Saraiya M. (2014). Centers for Disease Control and Prevention (CDC). Human papillomavirus vaccination: recommendations of the Advisory Committee on Immunization Practices (ACIP). *Morbidity and Mortality Weekly Report*.

[25] The NHS Choices (2014). *Vaccination. Cervical Cancer Vaccine*.

[26] Muñoz N., Kjaer S. K., Sigurdsson K. (2010). Impact of human papillomavirus (HPV)-6/11/16/18 vaccine on all HPV-associated genital diseases in young women. *Journal of the National Cancer Institute*.

[27] Lehtinen M., Paavonen J., Wheeler C. M. (2012). HPV PATRICIA Study Group. Overall efficacy of HPV-16/18 AS04-adjuvanted vaccine against grade 3 or greater cervical intraepithelial neoplasia: 4-year end-of-study analysis of the randomised, double-blind PATRICIA trial. *The Lancet Oncology*.

[28] The GlaxoSmithKline Vaccine HPV-007 Study Group (2009). Sustained efficacy and immunogenicity of the human papillomavirus (HPV)-16/18 AS04-adjuvanted vaccine: analysis of a randomised placebo-controlled trial up to 6.4 years. *The Lancet*.

[29] Konno R., Tamura S., Dobbelaere K., Yoshikawa H. (2010). Efficacy of human papillomavirus type 16/18 AS04-adjuvanted vaccine in Japanese women aged 20 to 25 years: final analysis of a phase 2 double-blind, randomized controlled trial. *International Journal of Gynecological Cancer*.

[30] Ault K. A., Future II Study Group (2007). Effect of prophylactic human papillomavirus L1 virus-like-particle vaccine on risk of cervical intraepithelial neoplasia grade 2, grade 3, and adenocarcinoma in situ: a combined analysis of four randomised clinical trials. *The Lancet*.

[31] Perez G., Lazcano P. E., Hernandez A. M. (2008). Safety, immunogenicity, and efficacy of quadrivalent human papillomavirus (types 6, 11, 16, 18) L1 virus-like-particle vaccine in Latin American women. *International Journal of Cancer*.

[32] Tay E. H., Garland S., Tang G. (2008). Clinical trial experience with prophylactic HPV 6/11/16/18 VLP vaccine in young women from the Asia-Pacific region. *International Journal of Gynecology and Obstetrics*.

[33] Majewski S., Bosch F., Dillner J. (2009). The impact of a quadrivalent human papillomavirus (types 6, 11, 16, 18) virus-like particle vaccine in European women aged 16 to 24. *Journal of the European Academy of Dermatology and Venereology*.

[34] Barr E., Gause C. K., Bautista O. M. (2008). Impact of a prophylactic quadrivalent human papillomavirus (types 6, 11, 16, 18) L1 virus-like particle vaccine in a sexually active population of North American women. *American Journal of Obstetrics & Gynecology*.

[35] de Carvalho N., Teixeira J., Roteli-Martins C. M. (2010). Sustained efficacy and immunogenicity of the HPV-16/18 AS04-adjuvanted vaccine up to 7.3 years in young adult women. *Vaccine*.

[36] Romanowski B. (2011). Long term protection against cervical infection with the human papillomavirus: review of currently available vaccines. *Human Vaccines*.

[37] Einstein M. H., Baron M., Levin M. J. (2009). Comparison of the immunogenicity and safety of Cervarix and Gardasil human papillomavirus (HPV) cervical cancer vaccines in healthy women aged 18–45 years. *Human Vaccines*.

[38] Einstein M. H., Baron M., Levin M. J. (2011). Comparative immunogenicity and safety of human papillomavirus (HPV)-16/18 vaccine and HPV-6/11/16/18 vaccine: follow-up from months 12-24 in a Phase III randomized study of healthy women aged 18-45 years. *Human Vaccines*.

[39] Barzon L., Squarzon L., Masiero S. (2014). Neutralizing and cross-neutralizing antibody titres induced by bivalent and quadrivalent human papillomavirus vaccines in the target population of organized vaccination programmes. *Vaccine*.

[40] Schiller J. T., Lowy D. R. (2009). Immunogenicity testing in human papillomavirus virus-like-particle vaccine trials. *Journal of Infectious Diseases*.

[41] Rambout L., Hopkins L., Hutton B., Fergusson D. (2007). Prophylactic vaccination against human papillomavirus infection and disease in women: a systematic review of randomized controlled trials. *Canadian Medical Association Journal*.

[42] Joura E. A., Kjaer S. K., Wheeler C. M. (2008). HPV antibody levels and clinical efficacy following administration of a prophylactic quadrivalent HPV vaccine. *Vaccine*.

[43] Lu B., Kumar A., Castellsagué X., Giuliano A. R. (2011). Efficacy and safety of prophylactic vaccines against cervical HPV infection and diseases among women: a systematic review & meta-analysis. *BMC Infectious Diseases*.

[44] Rey-Ares L., Ciapponi A., Pichon-Riviere A. (2012). Efficacy and safety of human papilloma virus vaccine in cervical cancer prevention: systematic review and meta-analysis. *Archivos Argentinos de Pediatria*.

[45] Traversa G., Sagliocca L., Liberati A., Martini N. (2010). The need to promote independent research on drugs. *Annals of Oncology*.

[46] Moher D., Liberati A., Tetzlaff J., Altman D. G., PRISMA Group (2009). Preferred reporting items for systematic reviews and meta-analyses: the PRISMA statement. *British Medical Journal*.

[47] Higgins J. P. T., Green S. (2014). *Cochrane Handbook for Systematic Reviews of Interventions Version 5.1.0*.

[48] Greenwood M., Yule G. U. (1915). The statistics of anti-typhoid and anti-cholera inoculations. *Proceedings of the Royal Society of Medicine*.

[49] Orozco-Colín A., Carrillo-García A., Méndez-Tenorio A. (2010). Geographical variation in human papillomavirus prevalence in Mexican women with normal cytology. *International Journal of Infectious Diseases*.

[50] del Amo J., González C., Belda J. (2009). Prevalence and risk factors of high-risk human papillomavirus in female sex workers in Spain: differences by geographical origin. *Journal of Women's Health (Larchmt)*.

[51] Muñoz N., Bosch F. X., Castellsagué X. (2004). Against which human papillomavirus types shall we vaccinate and screen? The international perspective. *International Journal of Cancer*.

[52] ClinicalTrails.Gov (2011). *Follow-up Study of GSK Biologicals' Human Papilloma Virus (HPV) Vaccine to Prevent Cervical Infection in Young Adults*.

[53] ClinicalTrails.Gov (2014). *Human Papilloma Virus (HPV) Vaccine Efficacy Trial Against Cervical Pre-cancer in Young Adults With GlaxoSmithKline (GSK) Biologicals HPV-16/18*.

[54] ClinicalTrails.Gov (2014). *Cervical Intraepithelial Neoplasm (CIN)-Warts Efficacy Trial in Women (Gardasil)*.

[55] ClinicalTrails.Gov (2014). *Cervical Intraepithelial Neoplasm (CIN) in Women (Gardasil)(V501-015)*.

[56] ClinicalTrails.Gov (2011). *Human Papillomavirus (HPV) Vaccine (Cervarix TM) Efficacy, Immunogenicity & Safety Trial in Adult Japanese Women With GSK Biologicals HPV-16/18 Vaccine*.

[57] Stanley M. (2007). Prophylactic HPV vaccines: prospects for eliminating ano-genital cancer. *British Journal of Cancer*.

[58] Alexander K. A., Giuliano A. R. (2012). HPV-beyond cervical cancer (online resource center). *The American Journal of Medicine*.

[59] Bosch F. X. (2011). Human papillomavirus: science and technologies for the elimination of cervical cancer. *Expert Opinion on Pharmacotherapy*.

[60] Haug C. J. (2008). Human papillomavirus vaccination—reasons for caution. *The New England Journal of Medicine*.

[61] Porta M., González B., Márquez S., Artazcoz L. (2008). Doubts on the appropriateness of universal human papillomavirus vaccination: is evidence on public health benefits already available?. *Journal of Epidemiology and Community Health*.

[62] Di Mario S., Basevi V., Borsari S., Balduzzi S., Magrini N. (2012). Overall efficacy of HPV-16/18 AS04-adjuvanted vaccine. *The Lancet Oncology*.

[63] Gravitt P. E. (2011). The known unknowns of HPV natural history. *Journal of Clinical Investigation*.

[64] Mattheij I., Pollock A. M., Brhlikova P. (2012). Do cervical cancer data justify HPV vaccination in India? Epidemiological data sources and comprehensiveness. *Journal of the Royal Society of Medicine*.

[65] Polzer J. C., Knabe S. M. (2012). From desire to disease: Human Papillomavirus (HPV) and the medicalization of nascent female sexuality. *Journal of Sex Research*.

[66] Safaeian M., Ghosh A., Porras C. (2012). Direct comparison of HPV16 serological assays used to define HPV-naïve women in HPV vaccine trials. *Cancer Epidemiology Biomarkers and Prevention*.

[67] Roteli-Martins C. M., Naud P., de Borba P. (2012). Sustained immunogenicity and efficacy of the HPV-16/18 AS04-adjuvanted vaccine: up to 8.4 years of follow-up. *Human vaccines & immunotherapeutics*.

[68] Lehtinen M., Apter D., Dubin G. (2006). Enrolment of 22,000 adolescent women to cancer registry follow-up for long-term human papillomavirus vaccine efficacy: guarding against guessing. *International Journal of STD and AIDS*.

[69] Jit M., Chapman R., Hughes O., Choi Y. H. (2011). Comparing bivalent and quadrivalent human papillomavirus vaccines: economic evaluation based on transmission model. *British Medical Journal*.

[70] Garattini L., van de Vooren K., Freemantle N. (2014). Tendering and value-based pricing: lessons from Italy on human papilloma virus vaccines. *Journal of the Royal Society of Medicine*.

[71] Liu B., Donovan B., Brotherton J. M. L., Saville M., Kaldor J. M. (2014). Genital warts and chlamydia in Australian women: comparison of national population-based surveys in 2001 and 2011. *Sexually Transmitted Infections*.

[72] Fairley C. K., Hocking J. S., Gurrin L. C., Chen M. Y., Donovan B., Bradshaw C. S. (2009). Rapid decline in presentations of genital warts after the implementation of a national quadrivalent human papillomavirus vaccination programme for young women. *Sexually Transmitted Infections*.

[73] Szarewski A., Skinner S. R., Garland S. M. (2013). Efficacy of the HPV-16/18 AS04-adjuvanted vaccine against low-risk HPV types (PATRICIA randomized trial): an unexpected observation. *The Journal of Infectious Diseases*.

[74] Kmietowicz Z. (2011). UK will use Gardasil in its HPV vaccination programme from next September. *The British Medical Journal*.

[75] Serrano B., Alemany L., Tous S. (2012). Potential impact of a nine-valent vaccine in human papillomavirus related cervical disease. *Infectious Agents and Cancer*.

[76] Joura E. A., Giuliano A. R., Iversen O. E. (2015). Broad Spectrum HPV Vaccine Study. A 9-valent HPV vaccine against infection and intraepithelial neoplasia in women. *The New England Journal of Medicine*.

[77] The PLOS Medicine Editors (2013). Getting more generous with the truth: clinical trial reporting in 2013 and beyond. *PLOS Medicine*.

[78] Godlee F. (2012). Clinical trial data for all drugs in current use. *British Medical Journal*.

