# Corrigendum to “Are the Two Human Papillomavirus Vaccines Really Similar? A Systematic Review of Available Evidence: Efficacy of the Two Vaccines against HPV”

**DOI:** 10.1155/2017/4583487

**Published:** 2017-05-30

**Authors:** Simona Di Mario, Vittorio Basevi, Pier Luigi Lopalco, Sara Balduzzi, Roberto D'Amico, Nicola Magrini

**Affiliations:** ^1^SaPeRiDoc Unit, Department of Primary Health Care, Regional Health Authority of Emilia-Romagna, Viale Aldo Moro 21, 40127 Bologna, Italy; ^2^Office of Chief Scientist, European Centre for Disease Prevention and Control (ECDC), 171 83 Stockholm, Sweden; ^3^Statistics Unit, Department of Diagnostic and Clinical Medicine and Public Health, University of Modena & Reggio Emilia, Via del Pozzo 71, 41100 Modena, Italy; ^4^Drug Evaluation Unit, WHO Collaborating Centre for Evidence Based Research Synthesis and Guidelines Development, Regional Health and Social Agency of Emilia-Romagna, Viale Aldo Moro 21, 40127 Bologna, Italy

In the article titled “Are the Two Human Papillomavirus Vaccines Really Similar? A Systematic Review of Available Evidence: Efficacy of the Two Vaccines against HPV” [1], there was an error in the Discussion section, where “Our systematic review highlights that, for precancerous lesions (CIN), the only available proxy of cervical cancer and heterogeneity among pooled studies is substantial” should be changed to “Our systematic review highlights that, for precancerous lesions (CIN), the only available proxy of cervical cancer, heterogeneity among pooled studies is substantial.” In addition, there was an error in Table 3. The corrected table is as follows.

## Figures and Tables

**Table 3:** Baseline characteristics of women enrolled in the four studies presented in the meta-analysis.

Study [reference]	Age mean (SD)		HPV 16 positivity at enrolment (%)				HPV 18 positivity at enrolment (%)				Lifetime number of sexual partners (median)			Smoking status (%)		Chlamydia trachomatis (%)		Hormonal contraceptive use (%)		Cytological abnormality at entry (%)	
			DNA		Serology		DNA		Serology												
	V	C	V	C	V	C	V	C	V	C		V	C	V	C	V	C	V	C	V	C
GSK [11, 12, 28]	20 ± 3	21 ± 3	NR	NR	NR	NR	NR	NR	NR	NR		NR	NR	NR	NR	NR	NR	NR	NR	NR	NR
PATRICIA [13, 27]	20 ± 3	20 ± 3	6	5	17	17	2	2	12	12	*0* ^∗^	4%	4%	30	30	6	5	59	61	10	9
											*1* ^∗^	74%	74%								
											*2* ^∗^	14%	15%								
											*≥3* ^∗^	8%	8%								
FUTURE I [16, 26, 30]	20 ± 2	20 ± 2	9	8	12	12	3	3	3	3		2	2	26	26	4	5	58	57	11	12
FUTURE II [17, 26, 30]	20 ± 2	20 ± 2	9	9	11	11	4	4	4	4		2	2	NR	NR	4	4	59	60	12	11

V: vaccine group; C: control group; NR: not reported. ^∗^Results are presented as percentage of women stratified by number of sexual partners.
