# Supplementary material for: Are the Two Human Papillomavirus Vaccines Really Similar? A Systematic Review of Available Evidence: Efficacy of the Two Vaccines against HPV
Source: J Immunol Res. 2015 Aug 25;2015:435141. doi: 10.1155/2015/435141 (PMC4562171; doi:10.1155/2015/435141)
Supplement: Supplementary file 1 — The supplementary material shows the search strategy that was adapted for each database searched. [file 435141.f1.pdf]

Annex 1 on-line. Search strategy.

(Papillomaviridae [MH] OR Alphapapillomavirus [MH] OR Human papillomavirus 16 [MH] OR Human papillomavirus 18 [MH] OR Human papillomavirus 6 [MH] OR Human papillomavirus 11 [MH] OR Papillomavirus Infections [MH] OR Uterine Cervical Neoplasms [MH] OR Cervical Intraepithelial Neoplasia [MH] OR Tumor Virus Infections [MH] OR Human papillomavirus)

AND

(Papillomavirus Vaccines [MH] OR human papillomavirus vaccine, L1 type 16, 18 OR human papillomavirus vaccine L1, type 6,11,16,18 OR HPV L1 protein, Human papillomavirus OR Viral Vaccines [MH] OR Capsid Proteins [MH] OR bivalent L1 virus-like-particle vaccine OR quadrivalent vaccine)
